# Long-term movements and activity patterns of platypus on regulated rivers

**DOI:** 10.1038/s41598-021-81142-6

**Published:** 2021-02-11

**Authors:** Tahneal Hawke, Gilad Bino, Richard T. Kingsford, Dion Iervasi, Kylie Iervasi, Matthew D. Taylor

**Affiliations:** 1 Centre for Ecosystem Science, School of Biological, Earth & Environmental Sciences, UNSW Sydney, Sydney, NSW 2052 Australia; 2 Austral Research and Consulting, PO Box 267, Port Fairy, VIC 3284 Australia; 3 NSW Department of Primary Industries – Fisheries, Port Stephens Fisheries Institute, Taylors Beach Rd, Taylors Beach, NSW 2316 Australia

**Keywords:** Ecology, Behavioural ecology, Freshwater ecology

## Abstract

The platypus is a semi-aquatic mammal, endemic to freshwater habitats of eastern Australia. There are gaps in the understanding of platypus movement behaviour within river systems, including spatial and temporal organization of individuals. We tracked movements of 12 platypuses on the regulated Snowy and Mitta Mitta Rivers for up to 12-months, the longest continuous tracking of platypus using acoustic telemetry. Platypuses remained relatively localized, occupying 0.73–8.45 km of river over 12 months, consistent with previous tracking studies over shorter periods. Males moved further than females, and larger males had higher cumulative movements, suggesting a possible relationship to metabolic requirements. Platypuses moved greater distances on the Mitta Mitta River, possibly associated with impacts of altered flow regimes to their macroinvertebrate diet. Increased movements and diurnal activity during winter were primarily driven by males, possibly attributable to breeding behaviours, rather than increased costs of winter foraging. Evidence for relatively small movements has implications for declining populations, given areas of localised declines are unlikely to be supplemented by migrating platypuses, especially when dispersal is restricted by dam walls. Understanding platypus movement behaviour is pertinent for their conservation, as water resource development and habitat modification continue to reduce connectivity between populations across their distribution.

## Introduction

Platypuses (*Ornithorhynchus anatinus*) occur mainly in creeks and rivers of eastern Australia, with an introduced population on Kangaroo Island^1^. They are primarily dependent on rivers and other water bodies, feeding exclusively on freshwater macroinvertebrates^2^, and using burrows on the water’s edge for resting and nesting^3^. Many aspects of platypus biology are increasingly well reported and studied, but movements, including spatial and temporal organisation of individuals, remains poorly understood^4^.

Linear home ranges of platypuses are typically < 15 km^4^, with sporadic long-distance movements^5^. Males generally have larger linear home ranges and move farther distances than females^3,6,7^, although lactating females may move greater distances^8^. There is overlap between male and female home ranges, reflecting their polygamous mating system^3,5,9,10^, with some males becoming territorial during breeding on some rivers^9,10^. Territorial behaviour generally occurs in late winter and is associated with increased testosterone and aggression^11,12^. Linear home ranges of juveniles are smaller than those of adults^3^, but male juveniles can move long distances during a dispersal phase^13^, as much as 44.4 km over a 30 week period^14^. Juveniles may be forced out of natal areas by adult resident platypuses^15^ or may disperse to reduce competition and avoid inbreeding^14^.

Platypuses are most active at night, but display diurnal activity on occasion^9,10,16^. They typically emerge from burrows within three hours of sunset^7^, with nightly foraging bouts spanning up to 11.3 km^8^. Foraging times also vary among individuals, particularly during breeding months^9^. Other evidence suggests that activity periods vary seasonally, with platypuses being more nocturnal during summer and autumn^16^ and diurnal activity increasing over the winter months^6,9^.

Long-distance, large-scale movements have been studied using mark-recapture surveys^10^, radiotelemetry^3,7,10^, data activity loggers^16^, and in-stream microchip readers^17^. However, mark-recapture surveys are labour intensive and recapture rates are low^14,18,19^, while other tracking methods (radiotelemetry, data activity loggers) are limited by battery life and attachment periods^17^, hindering continuous monitoring over long periods. Continuous long-term platypus tracking is essential for providing insights into changes in their movement behaviour. Acoustic transmitters can successfully track platypuses with externally attached transmitters, but there are attachment limitations^20–22^. Recently, intraperitoneal implants of acoustic transmitters have been used to provide data for up to six months (dependent on battery life^6^), suggesting this is an effective method for long-term continuous tracking of platypus movement.

In this study, we examined movement behaviour of platypuses on the regulated Snowy River (New South Wales) and the Mitta Mitta River (Victoria), using implanted acoustic transmitters and a fixed array of VR2W receivers. We investigated large-scale movements and present the longest continuous acoustic tracking time-series for individual platypuses yet reported. Data are used to evaluate differences in range, cumulative movements, and activity patterns among individuals in relation to demographic population structure and regulated river flows. We expected that males would have larger ranges and cumulative movements, particularly during the breeding season^3,6,7^. We also anticipated that juvenile males would move large distances during the study period, reflecting a potential dispersal phase^13^. Movements were expected to differ between rivers, reflecting differences in habitat, resources, and abundances.

## Methods

### Study sites

**Figure 1:**
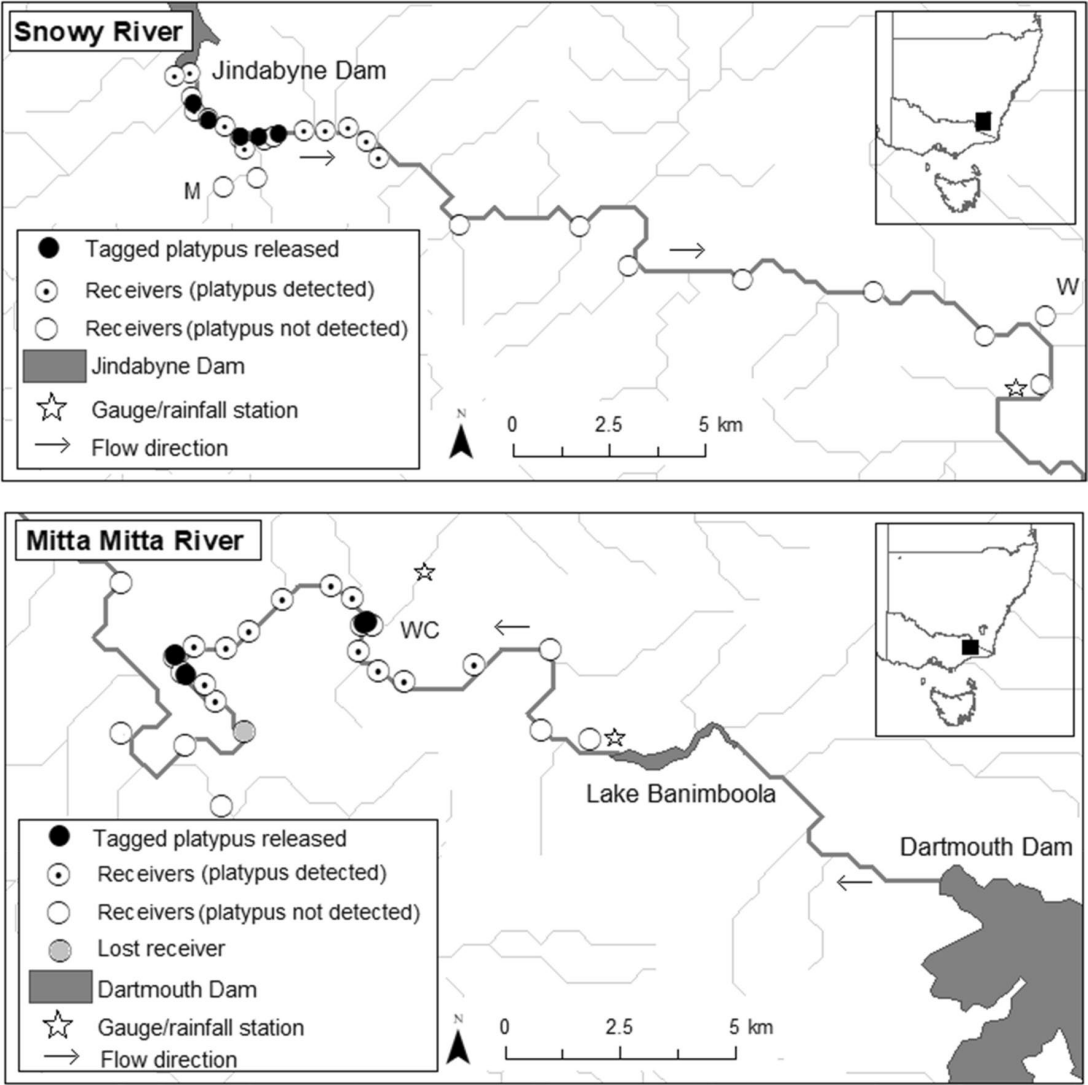
Locations of sites where acoustic transmitters were implanted in platypus, and locations of acoustic receivers used to detect their movements along the Snowy River (including two tributaries: M = Mowamba River and W = Wullwye Creek) and Mitta Mitta River (including one tributary: WC = Watchingorra Creek).

Platypus movements were investigated on the Snowy River, below Jindabyne Dam in south-eastern New South Wales and the Mitta Mitta River, below Dartmouth Dam in Victoria, Australia (Fig. 1). The Snowy River is characterised by deep pool and riffle sequences^23^, flowing through native eucalypt woodland (Eucalyptus spp.) in the Jindabyne Gorge and downstream adjoining grazing land^24^. It is regulated by the Jindabyne Dam, now receiving just 21% of its historic mean annual flow, increased from 1% after regulation^25^. Flows generally mimic the natural flow regime, with high flows over the spring (Stewardson & Gippel 2003). The Mitta Mitta River flows through a forested rocky gorge, emerging into cleared grazing land^26^. Downstream of Dartmouth Dam, the river is heavily regulated, with high summer flows diverted for irrigation^27^.

### Trapping

We captured platypuses using fyke nets or unweighted mesh (gill) nets. Fyke nets (30 mm knotless 20 ply nylon, 1 m × 5 m wings and 0.8 m × 5 m wings) were used in streams with depths < 1 m and set in pairs, facing upstream and downstream to capture animals moving in both directions. Fyke net cod (distal) ends were tied to stakes, ensuring nets sat at least 30 cm above the water level, allowing platypuses to breathe. Fyke nets were set in the late afternoon and checked 3-hourly until sunrise. Mesh nets (80 mm multifilament nets, 25 m × 2 m) were used in pools usually with depths > 2 m and lengths ≥ 50 m, between dusk and 01:00 h. Nets were spotlighted every 2–3 min for platypus and physically examined hourly to remove possible snags. Platypuses were removed from nets and transferred to pillowcases for around 30 min prior to tagging. We captured platypuses at five sites on the Snowy River, and three sites on the Mitta Mitta River. On both rivers we attempted to have some platypus capture sites as close to the dam walls as possible, to ascertain the impact of regulated flows to platypus movement. Trapping and handling of platypuses was carried out in accordance with guidelines and approved by the NSW Office of Environment and Heritage (SL101655), NSW Department of Primary Industries (P15/0096-1.0 & OUT15/26392), and UNSW’s Animal Care and Ethics Committee (16/14A).

### Movements

We used acoustic transmitters (Vemco Limited, Nova Scotia, Canada) and receivers (VR2W-069 k) to track platypus on the Snowy and Mitta Mitta Rivers. Acoustic transmitters broadcast a series of sound pulses, unique to each transmitter, which are detected by receivers placed in the water. On the Snowy River, implanted acoustic transmitters (V7-4L, 22.5 mm × 7 mm, weight in air 1.8 g, weight in water 1.0 g; 0.40% of average (± SD) juvenile female weight 0.46 ± 0.01 kg, 0.36% average juvenile male weight 0.50 ± 0.04 kg, 0.23% average female adult weight 0.80 ± 0.07 kg, 0.13% of average male adult weight 1.34 ± 0.16 kg; Table 1) were inserted in the peritoneal cavity of ten platypuses using anaesthesia, under established protocols reported in Bino et al. 2018. Platypuses were anaesthetised in the field using isoflurane (Pharmachem 5%) in oxygen (3 L/min) in an induction chamber, and then maintained using a T-piece facemask (1.5%, 1.0 L/min)^28^. To insert the transmitters, a small area of fur was removed from the ventral midline (5 mm × 15 mm longitudinally), halfway between the xiphisternum and the pubis. Prior to incision, 70% methanol was applied three times to the surgical site, followed by diluted chlorhexidine solution (0.1% weight/volume aqueous solution). A paper drape (5 mm × 15 mm) was attached and secured with glue at its edges. A 10 mm incision was made down to the linea alba, followed by an 8 mm incision into the peritoneum, both using a size-15 scalpel blade. The transmitter was flushed with sterile saline solution and then inserted into the peritoneal cavity, before the linea alba incision was closed with 3–4 single interrupted sutures. A few drops of bupivacaine hydrochloride were used as a local anaesthetic before the skin wound was closed with 1–2 cruciate sutures. Tissue adhesive (Vetbond) was also used to seal the incision. Platypuses were left to recover for a minimum of one hour before being returned to the water. Transmitters had a battery life expectancy of 197 days (~ 6.5 months).

**Table 1:** Identifications, weight (kg), detection dates, number of detections, maximum upstream (US) and downstream (DS) detection (km) from initial tagging location, and linear river range (km) for individual platypuses with transmitters on the Snowy River (Feb–Aug 2017) and Mitta Mitta River (May 2018–Apr 2019).

River	Platypus ID	Sex/age	Weight (kg)	Detection period	Detections (n)	Days detected	US/DS (km)	Range (km)
Snowy	FA1	Female/adult	0.72	1/03/17–1/08/17	1686	154	0.25/1.26	1.50
	FA2	Female/adult	0.89	2/03/17–1/08/17	1430	153	0/0.98	0.98
	FA3	Female/adult	0.78	2/03/17–31/07/17	2733	152	0/0.98	0.98
	FJ1	Female/juvenile	0.45	27/02/17–1/08/17	531	156	0.95/0.59	1.54
	FJ2	Female/juvenile	0.46	Not detected	0	0	0/0	0
	MA1	Male/adult	1.29	25/02/17–1/08/17	1713	158	0.95/2.66	3.61
	MA2	Male/adult	1.52	25/02/17–30/07/17	5554	156	0.95/3.80	4.76
	MA3	Male/adult	1.21	28/02/17–21/07/17	270	144	0.00/1.68	1.68
	MJ1	Male/juvenile	0.47	26/02/17–27/07/2017	24	152	0.33/0.40	0.73
	MJ2	Male/Juvenile	0.52	Not detected	0	0	0/0	0
Mitta Mitta	MA12	Male/adult	1.99	16/05/18–4/01/19	3761	234	3.24/1.83	5.07
	MA13	Male/adult	1.58	15/05/18–15/03/19	3180	305	3.24/5.21	8.45
	MA14	Male/adult	1.64	14/05/18–23/04/19	6079	345	0.57/1.27	1.84
	MA15	Male/adult	1.36	18/05/18–28/09/18	3516	134	1.51/0.90	2.41

To track implanted platypuses, 25 acoustic receivers were deployed along a 27 km stretch of river (February–August 2017, Fig. 1). Two receivers were placed above the Jindabyne Dam wall to ensure platypuses were detected if they were able to traverse the regulatory structure. Twelve receivers were concentrated for the first 5.5 km downstream of Jindabyne Dam wall (350–650-m intervals, average 475 ± 117 m), where the greatest numbers of platypus were trapped. The remaining receivers were located downstream at 3-km intervals (average 3 ± 0.23 km) to detect downstream long-distance movements and dispersal. Three receivers were placed on the Mowamba River tributary (350 m, 1.35 km and 2.35 km upstream from the junction with the Snowy River), and one receiver on the Wullwye Creek tributary (500 m from junction). Eight out of the ten platypuses with implanted transmitters were successfully detected and tracked over a 168-day period from February–August 2017 (Table 1). Two platypuses, (one juvenile male and juvenile female) failed to be detected after transmitters were implanted. Both platypuses were released into pools with a receiver, suggesting transmitter failure rather than an adverse outcome.

Despite considerable trapping effort on the Mitta Mitta River, only four platypuses were captured and tagged, with these captures not occurring directly downstream of the dam (minimum 8.4 km downstream; Fig. 1). These individuals were tagged with larger transmitters (V9-2L, 29 mm × 9 mm, weight in air 4.5 g, weight in water 2.7 g; 0.27% of average adult male weight 1.64 ± 0.26 kg; Table 1), which provided ~ 400 days (~ 13 months) of battery life. Twenty-five receivers were deployed to track the platypuses along a 32-km section of river below the Banimboola regulating pond (Fig. 1). A receiver was placed at each capture site, and two receivers placed at 500-m intervals upstream and downstream of the capture site (average 500 ± 100 m). One receiver was placed on Watchingorra Creek, 250 m upstream of the junction with the Mitta Mitta River. Remaining receivers were placed at 500-m–2-km intervals (average 1.4 ± 0.6 km), except for two receivers placed 5 km and 8.8 km further downstream to capture downstream large-scale movements or dispersal (Fig. 1). One receiver was lost during an extremely high flow event. All four platypuses were detected and tracked on the Mitta Mitta River.

### Statistical analysis

We tested for variation in range and cumulative movements of platypuses among months (March–May) and between sexes. Daily range was calculated as the distance along the river between the most upstream and downstream detections by any receiver, measured along the middle of the river. Daily cumulative movements were calculated as the sum of distances between two consecutive detections between two receivers. We tested for differences in range and cumulative movements using generalised mixed-effects models (GLMMs), using a gamma error distribution with a log link^29^. As the gamma distribution requires positive values, we added 50 m to all measured distances, aligning with detections uncertainties of acoustic receivers for this species^6,8,20,22^. Prior to analysis, juvenile platypuses (Table 1) and all data from February and August on the Snowy River were removed, given limited number of detections (203 records from five individuals and 11 detections from four platypuses, respectively). We used the ‘glmer’ function in the ‘lme4’ package^30^ in the R environment^31^. We considered sex (factor with two levels) and month (factor with five levels), as well as the interaction between sex and month, as the predictors on the Snowy River, while we considered only month on the Mitta Mitta River, as only males were captured (Table 1). For both rivers, the identity of tagged platypuses was included as a random effect, given we aimed to make conclusions regarding the larger population, from which these individuals were a random sample^6^. We used post-hoc tests for differences between months based on the estimated marginal means, using the ‘emmeans’ package^32^ in the R environment^31^.

We also tested for any effects of flow rate (ML/d), rainfall amount (mm), and number of records on daily range and cumulative movements. Flow rates and rainfall for the Snowy River were collected from Dalgety Weir Gauge (222,026, 36.51° S, 148.83° E; Fig. 1) (BOM, NSW Water). Flow rates for the Mitta Mitta River were collected at Coleman’s Gauge (582,010, 36.53° S, 147.46° E), and rainfall data were from Callaghan Creek Station (36.45° S, 147.43° E). All flow rates were natural-log transformed to reduce skewness and improve normality. We excluded all data from February and August due to low numbers of detections. We used Generalized Additive Mixed Models (GAMMs), offering a compromise between a linear model and a smoothing function, making them more flexible with fewer assumptions^33^. In GAMM models, splines are a collection of simple polynomial functions that are joined in locations known as knots. The flexibility of the spline curve is therefore determined by the number of knots specified in the model. Large numbers of knots can cause over-fitting of the model and so to avoid this, we limited the number of knots in the GAMMs to three^34^. We used the ‘gamm’ function in the ‘gamm4’ package^35^, in the R environment^31^. Similarly to the GLMMs, we used a gamma log link distribution and added 50 m to estimated distances. Daily range or cumulative movements were included as the response variable, and number of records, month (continuous), sex (two levels), flow rate (ML/d) and rainfall (mm) were included as predictors. We also included individual platypuses as a random effect, and an interaction term between sex and month to explore variation in monthly movements between sexes.

To investigate variation in hourly activity patterns, we calculated the percentage of nocturnal records, defined as those that occurred between one hour before sunset and one hour after sunrise. Average sunset/sunrise times were calculated from Jindabyne for the Snowy River and Albury for the Mitta Mitta River.

## Results

### General observations

Number of tracking days for the eight detected individuals on the Snowy River ranged from 144–158 (average 153 days). Number of detections ranged from 24 to 5554 (adult female average (± sd) 1950 ± 690, adult male average 2512 ± 2731, juvenile female 531, juvenile male 24) (Table 1). Platypuses were detected at 13/25 receivers and were not detected by receivers beyond 8.9 km downstream of the dam wall (Fig. 1). On the Mitta Mitta River, platypuses were detected for 134–345 days (average 255 days). Number of detections ranged from 3180 to 6079 (average 4134 ± 1318), with platypus being detected at 16/25 receivers (Fig. 1).

### Range movements

**Figure 2:**
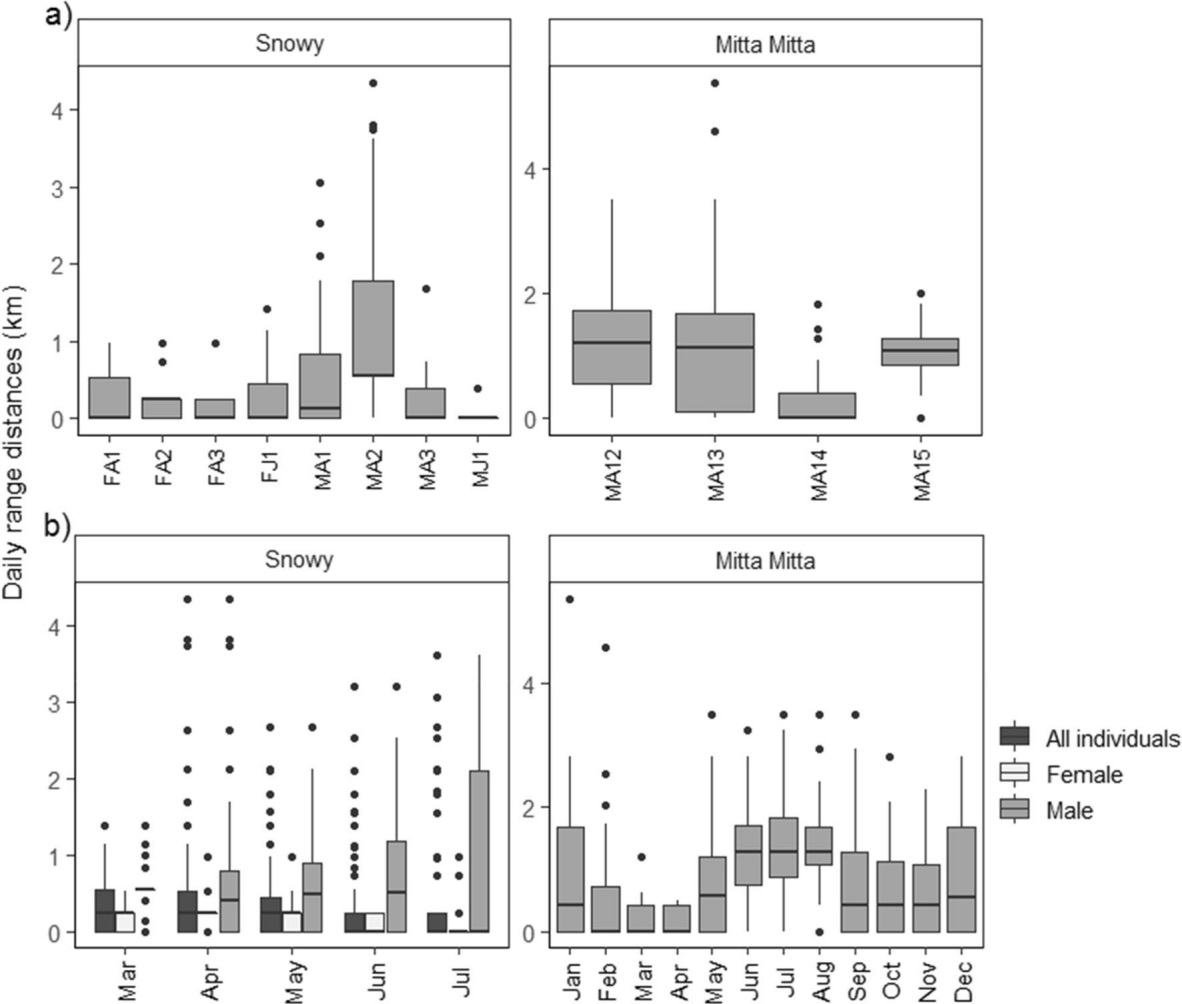
Daily river ranges (km) for platypuses on the Snowy River and Mitta Mitta River for (**a**) individuals across the entire study period and (**b**) monthly variation between sexes.

Most platypuses were detected both upstream and downstream of their initial tagging location (Table 1), with river position varying for each individual over time (Appendix 1). On the Snowy River, eight platypuses with implanted transmitters utilised between 0.73 and 4.76 km of river (Fig. 2a; Table 1; Appendix 1). The average daily range for all individuals was 0.39 ± 0.66 km. MA2 had the highest average (1.10 ± 1.04 km) and maximum (4.35 km) daily river range and was detected over a total linear river range of 4.76 km. The male juvenile (MJ1) had the smallest movement range of tracked platypuses, with a range of 730 m over 151 days, but was detected far less often than other platypuses in the study (Table 1). The average daily range for male adults was greater than for female adults, significantly higher in all months (*P* ≤ 0.020) except March (*z* = − 1.554, *P* = 0.120) and April (*z* = − 1.461, *P* = 0.144; Fig. 2b, Appendix 2). For females, average daily ranges were significantly higher in April (0.26 ± 0.19 km) compared to all other months (*P* ≤ 0.041) and significantly lower in June (0.07 ± 0.11 km) compared to all other months (*P* ≤ 0.001) except July (0.12 ± 0.25 km, *z* = − 1.689, *P* = 0.072), (Fig. 2b; Appendix 2). For males, movement ranges peaked in the winter month of July (0.94 ± 1.22 km), significantly higher than March and June (*P* ≤ 0.049) and marginally higher compared to April and May (*P* ≤ 0.093), (Fig. 2b; Appendix 2). Based on GAMM models, daily range of platypus movements had a significant positive association with number of detections up to 100 detections but declined thereafter (F = 16.911, *P* < 0.001), a significant decreasing effect of flow rates (ML/d) (F = 4.343, *P* = 0.037; Appendix 2) and no associations with rainfall (mm) (F = 0.506, *P* = 0.477).

On the Mitta Mitta River, the daily average range of the four males was 1.0 ± 0.46 km, with platypuses utilising 1.84–8.45 km of river over the 12-month period. MA12 and MA13 had far greater movements upstream and downstream, compared to MA14 and MA15 (Fig. 2a; Table 1). MA12 had the highest average daily range (1.39 ± 1.00 km), while MA13 had the highest maximum daily range (3.50 km) and used a linear river range of 8.45 km during the 12-month period. Range movements were significantly higher in June (1.26 ± 0.75 km), July (1.46 ± 0.91 km), and August (1.37 ± 0.75 km), compared with all other months (*P* ≤ 0.001; Fig. 2b, Appendix 2). Based on GAMM models, daily range movements had a significant positive association with number of detections (F = 48.919, *P* < 0.001, Appendix 2), and flow (F = 11.899, *P* < 0.001, Appendix 2), increasing towards 1900 ML/d, but decreasing with greater flow rates. No association were detected with rainfall (F = 0.946, *P* = 0.331, Appendix 2).

### Cumulative movements

**Figure 3:**
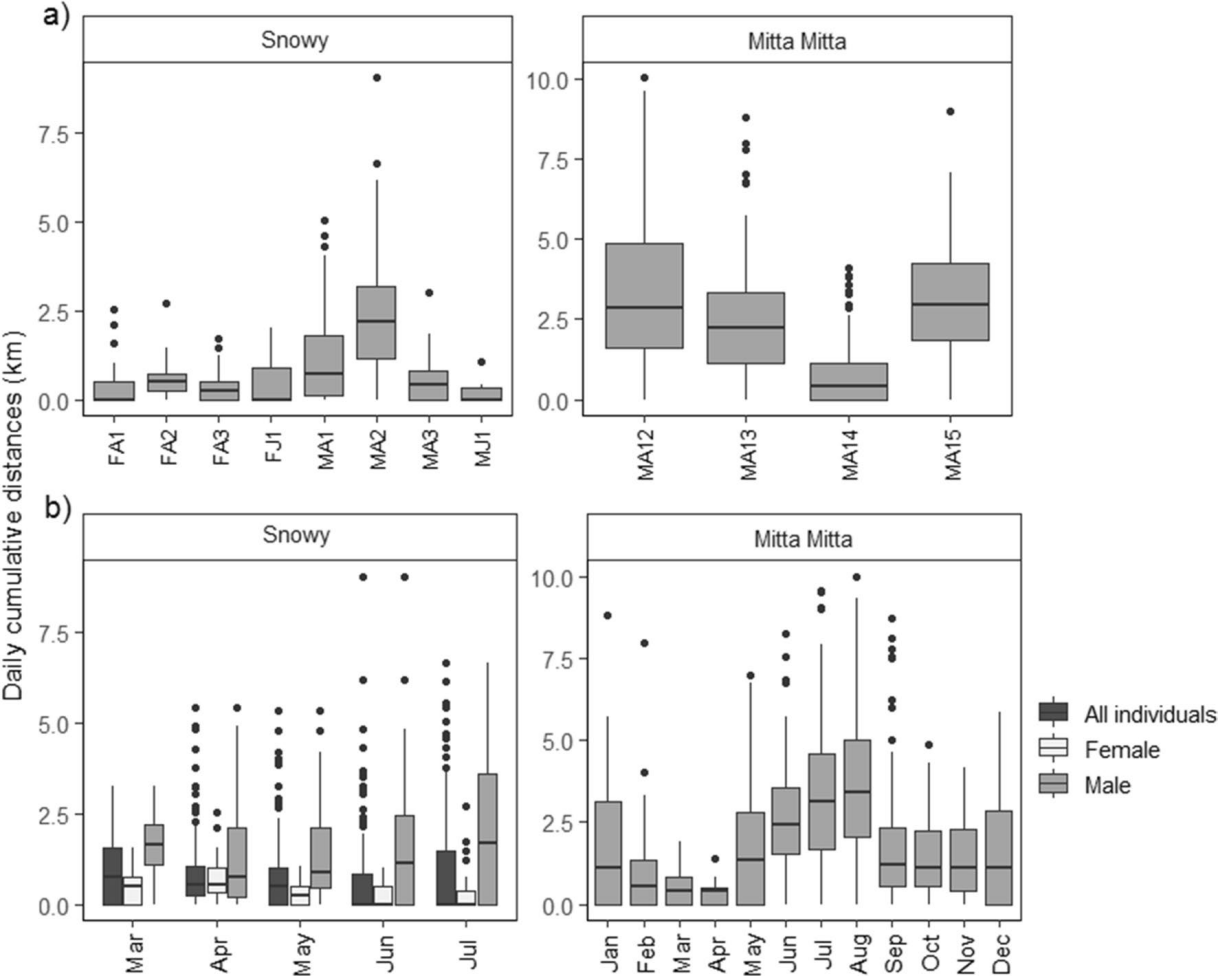
Daily cumulative movements (km) for platypuses on the Snowy River and Mitta Mitta River for (**a**) individuals across the entire study period and (**b**) monthly variation between sexes.

On the Snowy River, average cumulative daily movements were 0.87 ± 1.18 km. MA2 had the highest average (2.37 ± 1.57 km) and maximum (9.04 km) daily cumulative movements and travelled 335.30 km over 155 days (Fig. 3a). Adult males had significantly higher cumulative daily movements compared to females for all months (*P* ≤ 0.004) except April (*z* = − 0.582, *P* = 0.560; Fig. 3b, Appendix 3). Cumulative daily movements of females were higher in April (0.65 ± 0.49 km) compared to all months (*P* ≤ 0.017) and significantly lower in June (0.19 ± 0.29 km) compared to all months (*P* ≤ 0.001) except July (0.27 ± 0.50 km, *z* = 1.400, *P* = 0.162). Male cumulative movements were significantly higher in July (2.03 ± 1.99 km) compared to April (1.30 ± 1.14 km, *z* = − 2.575, *P* = 0.010), and marginally higher than in March, May, and June (*P* ≤ 0.085; Appendix 3). Based GAMM models, the number of detections was positively associated with range up to 100 detections but declined thereafter (F = 21.398, *P* < 0.001), a negative association with flow (F = 4.254, *P* = 0.029), and no association with rainfall (F = 0.052, *P* = 0.820), (Appendix 3).

On the Mitta Mitta River, average cumulative daily movements were 2.43 ± 1.22 km. MA12 had the highest average (3.48 ± 2.46 km) and maximum (9.61 km) daily cumulative movements and travelled 779.14 km over the 12-month period (Fig. 3a). Cumulative movements were significantly higher in June (2.75 ± 1.75 km), July (3.44 ± 2.23 km), and August (3.73 ± 2.12 km), compared with all other months (*P* ≤ 0.001; Fig. 3b, Appendix 3). Based on GAMM models, cumulative daily movements were significantly associated with number of detections (F = 61.704, *P* < 0.001). Cumulative daily movements had a positive association with flow, increasing towards 1900 ML/d, before a slight decrease (F = 24.124, *P* < 0.001) and a significant positive association with rainfall (F = 5.235, *P* = 0.022; Appendix 3).

### Activity patterns

**Figure 4:**
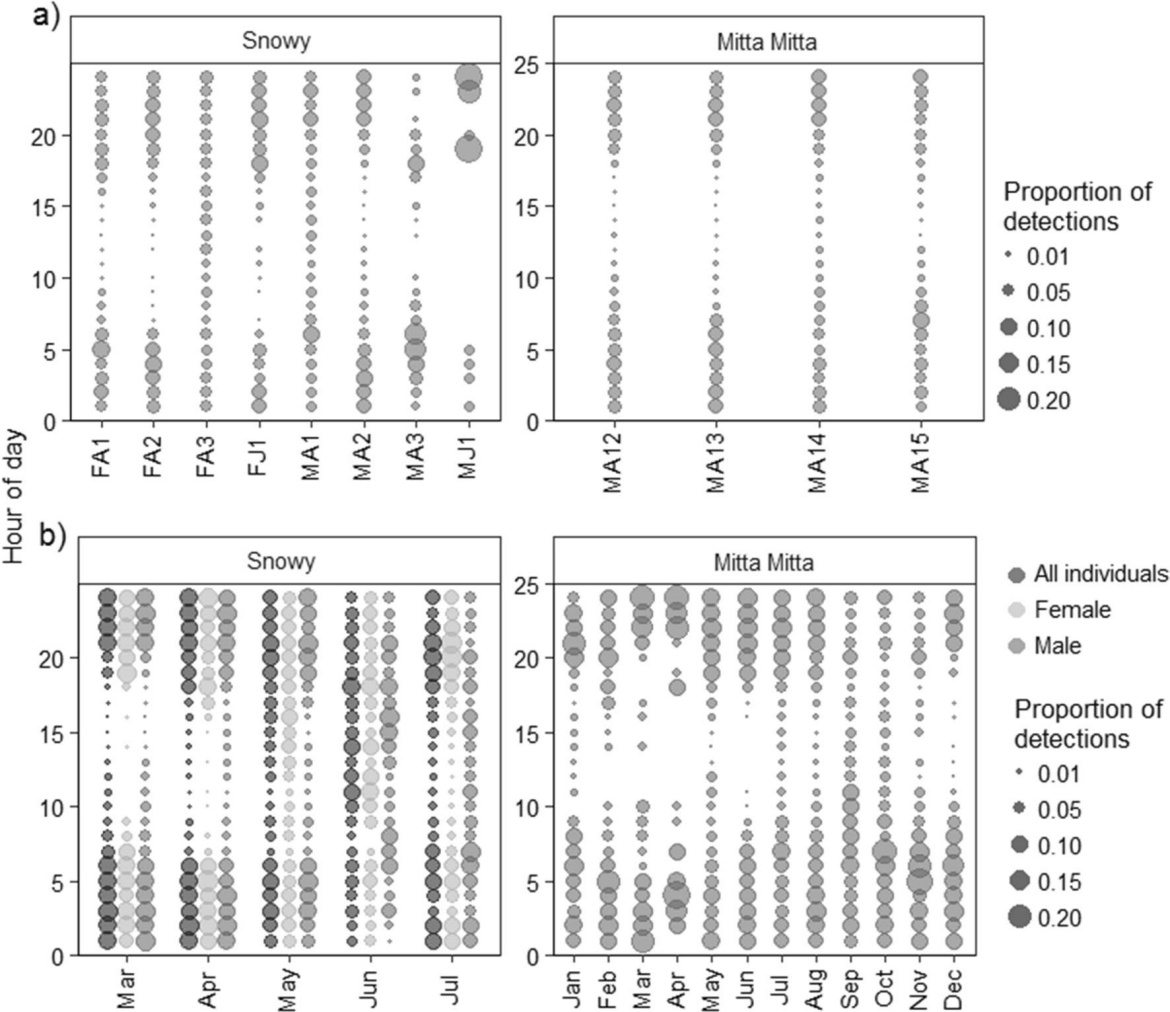
Proportion of hourly detections for platypuses on the Snowy River ad Mitta Mitta River for (**a**) individuals across the entire study period and (**b**) monthly variation between sexes.

On the Snowy River, platypuses varied their daily activity patterns each month (Fig. 4, Table 2). Platypuses displayed mostly primarily nocturnal activity. This was most pronounced in April, with 93.2% of detections between one hour before sunset and one hour after sunrise (henceforth ‘night’) (average time of sunset 17:37, average time of sunrise 06:31). Nocturnal activity was less common during June, with 67.8% of detections at night (15:56, 08:15). There was variation between sexes, with females exhibiting more nocturnal behaviour than males during March, April, and July (Table 2).

**Table 2:** Average times one hour before and after sunset and percentage of nocturnal platypus records (between one hour before sunset and one hour after sunrise) on the Snowy River (March–July 2017) and Mitta Mitta River (May 2018–April 2019).

River	Month	Sunset (− 1 h)	Sunrise (+ 1 h)	Percentage of records between sunset (− 1 h) and sunrise (+ 1 h)	Females (%)	Males (%)
Snowy	March	18:20	8:05	90.9	95.6	89.3
	April	16:37	7:31	93.2	97.7	89.7
	May	16:06	7:56	80.3	74.2	90.3
	June	15:56	8:15	67.8	67.0	70.4
	July	16:08	8:13	86.3	93.0	72.0
Mitta Mitta	May	16:14	8:02	89.0		
	Jun	16:04	8:20	98.6		
	Jul	16:16	8:19	86.3		
	Aug	16:38	7:53	90.9		
	Sep	17:01	7:12	60.4		
	Oct	18:15	7:17	71.4		
	Nov	18:56	6:57	79.3		
	Dec	19:23	6:51	82.8		
	Jan	19:28	7:13	78.5		
	Feb	19:06	7:43	80.9		
	Mar	18:28	8:11	88.2		
	Apr	16:58	7:49	95.3		

On the Mitta Mitta River, platypuses were most nocturnal in June (98.6% of detections at night; 17:07, 7:20) and April (95.3% of detections between 17:58, 6:49), but nocturnal behaviour was prominent for most months (Table 2). Platypuses were least nocturnal during the spring months, particularly in September (60.4% of detections at night; 17:01 and 7:12).

## Discussion

Tracking movements of mobile and aquatic animals is critical for understanding their life history and behaviour, resulting in better management of their threats^36–38^, and how they respond to changes in environmental variables^39^. We tracked movements of twelve platypuses on the Snowy and Mitta Mitta Rivers, using implanted acoustic transmitters for up to 12-months. There were consistencies between movement behaviour in this study and previously reported movements for platypuses, but range, cumulative movements, and activity also varied between sexes, among months, and potentially in response to river regulation. There was also some variation between rivers, likely reflecting differences in social organisation, competition, habitat, and the availability of local resources^5,7,9,10,40^.

Similar to previous studies, platypuses were relatively localised, with their movements, remaining within a few kilometres of their initial tagging location, despite the longer period over which we monitored individuals. Previously, studies suggested the maximum linear range male platypuses effectively patrol to be around 7 km, resulting in a maximum movement of 14 km in a 24-h period for a return trip^5^. Although our sample size was small, this was similar to our maximum linear range of 8.45 km, and distances of 0.5–15 km for tracked platypuses on other rivers^4^. Movement, site fidelity, and home range in mammals is influenced by accessibility of mates^41^ and resources^42^. Site fidelity may also be beneficial in populations with male–male conflict, with resident males shown to have an advantage over intruders^43,44^. Platypuses likely confine themselves to these ranges because mates and adequate food sources are available.

While there were some consistencies in movement, there was also variation, particularly between sexes and age groups. Females moved less than males on the Snowy River and were not detected more than 1.5 km from their initial tagging location, consistent with platypus behaviour on other rivers^3,7^. Longer movements in males is likely due to territorial and mate acquisition requirements during the breeding season^14,16^, but we also identified these patterns over the non-breeding months (Fig. 4b). Interestingly, the largest males moved furthest on the Snowy River and Mitta Mitta River (Figs. 2a, 3a), indicating some possible positive relationship between increasing body size and movements, not apparent from the Goulburn River, Victoria^9^, although this could not be tested in this study due to the low sample size of individuals. The relationship between increasing movements and body mass occurs in different mammals^45^, birds and lizards^46^, and turtles^47^. It may be related to higher energetic requirements in larger animals^48^ and the need to forage more widely^14^.

Despite high sampling effort, no juveniles were tagged on the Mitta Mitta River, and two of the four tagged juveniles on the Snowy River could not be tracked, limiting our capacity to assess juvenile dispersal. In mammals, dispersal tends to be male-based^49^, with juvenile males of many species dispersing away from the natal area after weaning^50,51^. Juvenile male platypuses can travel long distances from natal sites^6,13,14^, which probably reduces competition and inbreeding, and facilitates access to new home ranges. We tracked two juveniles on the Snowy River, with the male juvenile (MJ1) travelling the smallest distances (Figs. 2a, 3a). MJ1 was detected at only a single receiver (660.13 m downstream of the Jindabyne Dam wall, Fig. 1), suggesting limited movements, before he moved 400 m downstream of his resident pool on a single day, during the final week of the study in July. Potentially, this might represent the initiation of his dispersal phase, but this cannot be robustly inferred from this study. Movements upstream were also probably not possible as he was captured within a kilometre of the Jindabyne Dam wall, likely impacting dispersal for this platypus^52,53^.

Platypuses also varied their movements with time of year, with differences between sexes and subtle differences between rivers. Platypuses generally move further during the winter months^16^, perhaps because of increased thermoregulatory costs (16–20% higher during winter) and reduced diversity and size of benthic organisms^54^. However, in this study males increased their movements over the winter months on the Snowy and Mitta Mitta Rivers, but both range and cumulative movements were lower in winter than in the autumn months for females (Figs. 2b, 3b). If platypuses were increasing their range and cumulative movements to counteract the costs of winter foraging, we would expect this to be consistent across sexes. Given increases were more likely for males, this may reflect increased efforts to establish territories and locate females prior to breeding^9^. Platypuses have also been shown to increase diurnal activity over the winter months^6,9^. In Tasmania, increased diurnal activity occurred primarily in females^16^, but males were more active in the day than females during July on the Snowy River, complementing increased movements in the lead up to the breeding season^4^.

While there were some similarities in movement and activity patterns on both rivers, there were also some notable differences. Average daily cumulative movements on the Mitta Mitta River were higher than on the Snowy River. These differences may reflect possible scarcity of habitat and resources downstream of Dartmouth Dam on the Mitta Mitta River. Due to extensive regulation, water temperatures downstream of Dartmouth Dam can be as much as 12 °C below normal^55^, which may reduce macroinvertebrate food availability downstream of the dam^26,56^, also reflected in reduced abundances of platypuses compared to capture rates on the Snowy River^57^. There was some evidence that flows were associated with both range and cumulative movements, suggesting that platypuses may reduce foraging distances under higher flows due to increased energetic demands^8^. Identifying how flows interact with prey abundance and platypus movements, remains a critical gap in understanding habitat requirements and estimating population sizes.

This study was limited by the small sample sizes (number of tracked platypuses) and limited replication (number of rivers), limiting the scope of inference about long-term movements of platypus, particularly in relation to sex and life stage. Although significant effort was made, the number of tracked platypuses was particularly small on the Mitta Mitta River, with only four males captured. The small numbers of tagged platypuses also limited comparisons between the two river systems, but this probably reflected the poor state of the platypus population on the Mitta Mitta River^57^. Movement tracking of platypuses on both rivers occurred for periods of 6–12 months, some of the longest movement tracking data for this species. However, this was still not long enough to adequately represent annual variation of movement behaviours in relation to breeding and resource availability. Some variation probably also occurred among rivers due to the fixed placement of acoustic receivers, potentially resulting in underestimation of ranges and cumulative movements of platypuses foraging in areas with no receivers. These limitations are inevitable when studying a cryptic freshwater species found in low densities.

Platypus movements are increasingly understood, particularly the existence of restricted ranges, after potential dispersal phases for male juveniles. This has implications for declining populations^58,59^, given that populations in areas of local declines and extinctions are unlikely to be supplemented by migrating platypuses. This is particularly problematic if dispersing juveniles are restricted by dam walls, potentially impacting metapopulation connectivity and the future viability of populations^60^. While there was no strong evidence of impacts of changing regulated flows to movements, platypus movements may be indirectly influenced by the impacts of river regulation on abundance of their macroinvertebrate prey. Future research would benefit from tracking platypuses to assess habitat use and quality, and the relationship this has with their macroinvertebrate food sources. Understanding platypus movements, particularly on regulated systems, is increasingly important for their conservation, given ongoing declines and drying of water bodies across their distribution.

## Supplementary Information

Supplementary Information.
