# Supplementary material for: Long-term movements and activity patterns of platypus on regulated rivers
Source: Sci Rep. 2021 Feb 11;11:3590. doi: 10.1038/s41598-021-81142-6 (PMC7878892; doi:10.1038/s41598-021-81142-6)

**Long term movements and activity patterns of platypus on regulated rivers**

Tahneal Hawke^1^*, Gilad Bino^1^, Richard T. Kingsford^1^, Dion Iervasi^2^, Kylie Iervasi^2^ Matthew D. Taylor^1,3^

^1^Centre for Ecosystem Science, School of Biological, Earth & Environmental Sciences, UNSW Sydney, 2052 NSW, Australia; [*t.hawke@unsw.edu.au](mailto:*t.hawke@unsw.edu.au)

^2^Austral Research and Consulting, PO Box 267, Port Fairy 3284, VIC, Australia.

^3^Port Stephens Fisheries Institute, NSW Department of Primary Industries – Fisheries, Taylors Beach Rd, Taylors Beach, New South Wales, 2316, Australia.

**Appendix**

***Appendix 1. River positions***

### Figure A1. River positions where platypuses were detected for a) platypuses with implanted transmitters on the Snowy River, b) platypuses on the Mitta Mitta River, measured in km from the dam wall.
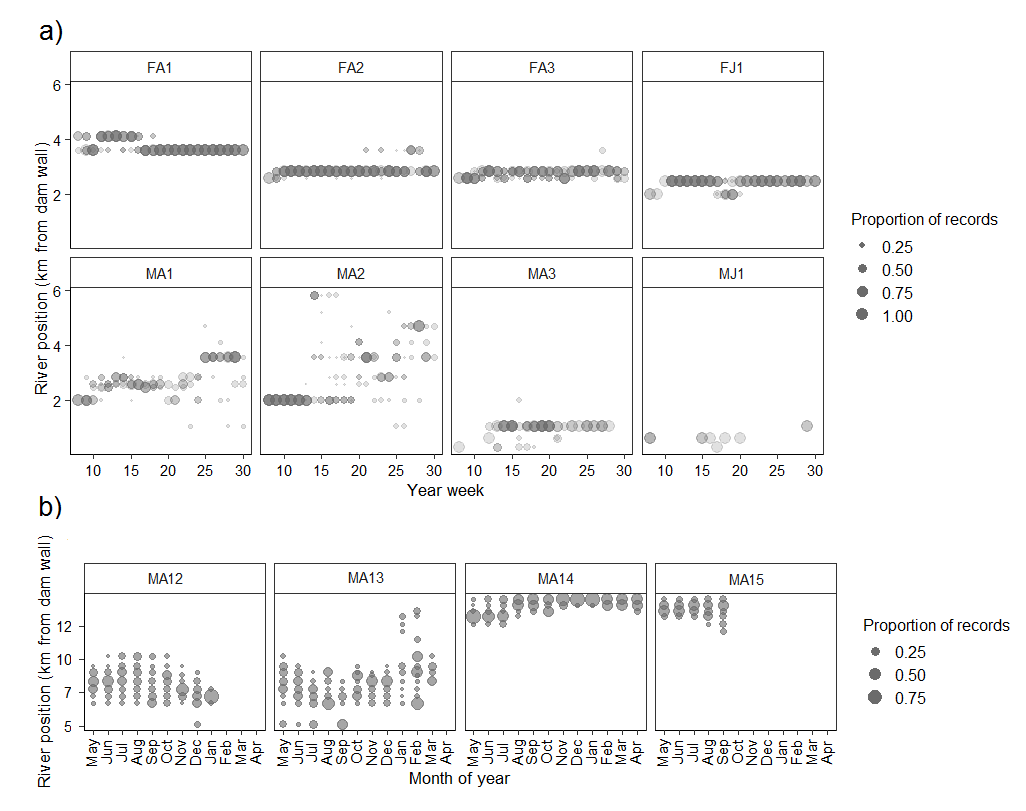


## Appendix 2. Models for range movements

### Table A1. Model coefficients of the Generalized Mixed Effect Model of daily ranges moved by platypuses with implanted transmitters on the Snowy River across months with an interaction between month and sex (Mar-Jul 2017).

| **Fixed effects** | **Estimate** | **Std. Error** | **t value** | **P** |
| --- | --- | --- | --- | --- |
| (Intercept) | -1.381 | 0.261 | -5.285 | <0.001 |
| April | 0.288 | 0.141 | 2.044 | 0.041 |
| May | -0.290 | 0.145 | -2.000 | 0.045 |
| June | -0.839 | 0.148 | -5.658 | <0.001 |
| July | -0.572 | 0.150 | -3.817 | <0.001 |
| Male | 0.590 | 0.380 | 1.554 | 0.120 |
| April:Male | -0.047 | 0.223 | -0.213 | 0.831 |
| May:Male | 0.522 | 0.234 | 2.229 | 0.026 |
| June:Male | 1.011 | 0.239 | 4.239 | <0.001 |
| July:Male | 1.114 | 0.233 | 4.779 | <0.001 |
| AIC BIC logLik deviance df.resid  -141.0 -85.5 82.5 -165.0 741 | | | | |

### Table A2. Post-hoc tests based on estimated marginal means of a Generalized Mixed Effect Model of daily ranges moved by platypuses with implanted transmitters on the Snowy River

| **Contrast** | **Estimate** | **SE** | **z.ratio** | **p.value** |
| --- | --- | --- | --- | --- |
| Mar Female - Apr Female | -0.288 | 0.141 | -2.044 | 0.041 |
| Mar Female - May Female | 0.290 | 0.145 | 2.000 | 0.046 |
| Mar Female - Jun Female | 0.839 | 0.148 | 5.658 | <.0001 |
| Mar Female - Jul Female | 0.572 | 0.150 | 3.817 | 0.000 |
| Mar Female - Mar Male | -0.590 | 0.380 | -1.554 | 0.120 |
| Mar Female - Apr Male | -0.831 | 0.371 | -2.238 | 0.025 |
| Mar Female - May Male | -0.822 | 0.373 | -2.204 | 0.028 |
| Mar Female - Jun Male | -0.763 | 0.383 | -1.992 | 0.046 |
| Mar Female - Jul Male | -1.132 | 0.380 | -2.977 | 0.003 |
| Apr Female - May Female | 0.578 | 0.146 | 3.964 | 0.000 |
| Apr Female - Jun Female | 1.127 | 0.149 | 7.561 | <.0001 |
| Apr Female - Jul Female | 0.861 | 0.151 | 5.702 | <.0001 |
| Apr Female - Mar Male | -0.302 | 0.380 | -0.795 | 0.427 |
| Apr Female - Apr Male | -0.543 | 0.372 | -1.461 | 0.144 |
| Apr Female - May Male | -0.534 | 0.373 | -1.431 | 0.153 |
| Apr Female - Jun Male | -0.475 | 0.383 | -1.239 | 0.215 |
| Apr Female - Jul Male | -0.844 | 0.380 | -2.218 | 0.027 |
| May Female - Jun Female | 0.549 | 0.144 | 3.820 | 0.000 |
| May Female - Jul Female | 0.282 | 0.144 | 1.958 | 0.050 |
| May Female - Mar Male | -0.881 | 0.379 | -2.321 | 0.020 |
| May Female - Apr Male | -1.121 | 0.371 | -3.024 | 0.003 |
| May Female - May Male | -1.112 | 0.373 | -2.986 | 0.003 |
| May Female - Jun Male | -1.053 | 0.382 | -2.754 | 0.006 |
| May Female - Jul Male | -1.422 | 0.380 | -3.746 | 0.000 |
| Jun Female - Jul Female | -0.266 | 0.148 | -1.802 | 0.072 |
| Jun Female - Mar Male | -1.429 | 0.381 | -3.754 | 0.000 |
| Jun Female - Apr Male | -1.670 | 0.372 | -4.486 | <.0001 |
| Jun Female - May Male | -1.661 | 0.374 | -4.443 | <.0001 |
| Jun Female - Jun Male | -1.602 | 0.384 | -4.175 | <.0001 |
| Jun Female - Jul Male | -1.971 | 0.381 | -5.173 | <.0001 |
| Jul Female - Mar Male | -1.163 | 0.381 | -3.052 | 0.002 |
| Jul Female - Apr Male | -1.403 | 0.373 | -3.767 | 0.000 |
| Jul Female - May Male | -1.395 | 0.374 | -3.726 | 0.000 |
| Jul Female - Jun Male | -1.335 | 0.384 | -3.477 | 0.001 |
| Jul Female - Jul Male | -1.704 | 0.381 | -4.470 | <.0001 |
| Mar Male - Apr Male | -0.241 | 0.173 | -1.394 | 0.163 |
| Mar Male - May Male | -0.232 | 0.184 | -1.262 | 0.207 |
| Mar Male - Jun Male | -0.172 | 0.187 | -0.923 | 0.356 |
| Mar Male - Jul Male | -0.542 | 0.178 | -3.035 | 0.002 |
| Apr Male - May Male | 0.009 | 0.156 | 0.057 | 0.955 |
| Apr Male - Jun Male | 0.068 | 0.177 | 0.385 | 0.700 |
| Apr Male - Jul Male | -0.301 | 0.173 | -1.734 | 0.083 |
| May Male - Jun Male | 0.059 | 0.187 | 0.318 | 0.751 |
| May Male - Jul Male | -0.310 | 0.184 | -1.680 | 0.093 |
| Jun Male - Jul Male | -0.369 | 0.188 | -1.966 | 0.049 |

### Table A3. Model coefficients of Generalized Additive Mixed Model of the association between average daily range movements by platypuses with implanted transmitters on the Snowy River, in response to month, flow and total number of detections, with an interaction term among individual platypuses and month.

| **Parametric coefficients** | **Estimate** | **Std. Error** | **T value** | **P** |
| --- | --- | --- | --- | --- |
| (Intercept) | -1.249 | 0.230 | -5.426 | <0.001 |
|  | | | | |
| **Smooth terms** | **edf** | **Ref.df** | **F** | **p-value** |
| s(counts) | 1.945 | 1.945 | 16.911 | <0.001 |
| s(month):Female | 1.000 | 1.000 | 12.903 | <0.001 |
| s(month):Male | 1.000 | 1.000 | 27.126 | <0.001 |
| s(rainfall) | 1.000 | 1.000 | 0.506 | 0.477 |
| s(Log_flow) | 1.000 | 1.000 | 4.343 | 0.037 |
| R-sq.(adj) = 0.0512 Scale est. = 1.2165 n = 875 | | | | |

### Figure A2. Generalized Additive Mixed Model plots of the average daily range movements by platypuses with implanted transmitters on the Snowy River.


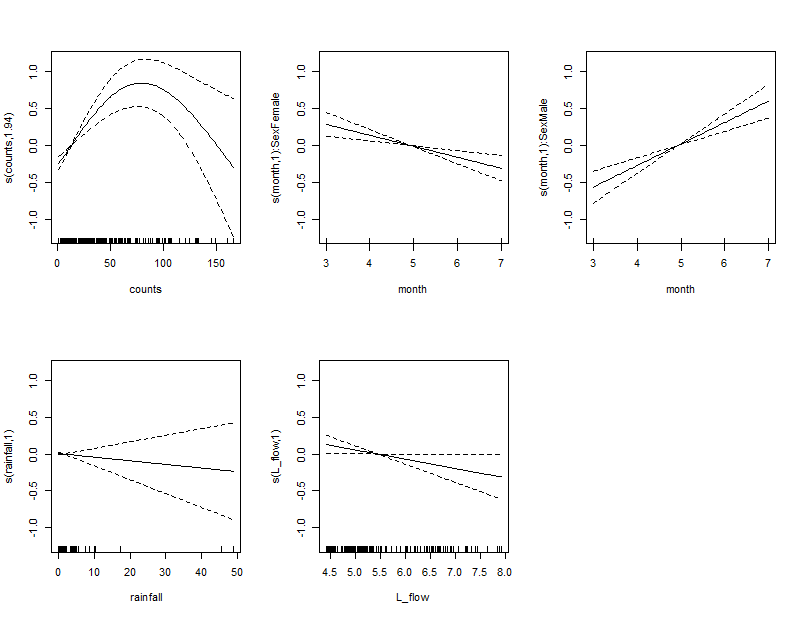


### Table A4. Model coefficients of the Generalized Mixed Effect Model of daily ranges move by platypuses with on the Mitta Mitta River across months (May 2018-Apri 2019).

| **Fixed effects** | **Estimate** | **Std. Error** | **t value** | **P** |
| --- | --- | --- | --- | --- |
| (Intercept) | -0.860 | 0.374 | -2.299 | 0.022 |
| August | 1.167 | 0.263 | 4.432 | <0.001 |
| December | 0.249 | 0.272 | 0.915 | 0.360 |
| February | 0.059 | 0.299 | 0.198 | 0.843 |
| January | 0.250 | 0.281 | 0.888 | 0.375 |
| July | 1.275 | 0.262 | 4.866 | <0.001 |
| June | 1.138 | 0.263 | 4.333 | <0.001 |
| March | -0.310 | 0.298 | -1.040 | 0.298 |
| May | 0.560 | 0.276 | 2.029 | 0.042 |
| November | 0.031 | 0.273 | 0.114 | 0.909 |
| October | 0.175 | 0.270 | 0.648 | 0.517 |
| September | 0.437 | 0.267 | 1.637 | 0.102 |
| AIC BIC logLik deviance df.resid  1413.4 1480.7 -692.7 1385.4 896 | | | | |

### Table A5. Post-hoc tests based on estimated marginal means of a Generalized Mixed Effect Model of daily ranges moved by platypuses with implanted transmitters on the Mitta Mitta River.

| **Contrast** | **Estimate** | **SE** | **z.ratio** | **p.value** |
| --- | --- | --- | --- | --- |
| Apr - Aug | -1.167 | 0.263 | -4.432 | <.0001 |
| Apr - Dec | -0.249 | 0.272 | -0.915 | 0.360 |
| Apr - Feb | -0.059 | 0.299 | -0.198 | 0.843 |
| Apr - Jan | -0.250 | 0.281 | -0.888 | 0.375 |
| Apr - Jul | -1.275 | 0.262 | -4.866 | <.0001 |
| Apr - Jun | -1.138 | 0.263 | -4.333 | <.0001 |
| Apr - Mar | 0.310 | 0.298 | 1.040 | 0.298 |
| Apr - May | -0.560 | 0.276 | -2.029 | 0.042 |
| Apr - Nov | -0.031 | 0.273 | -0.114 | 0.909 |
| Apr - Oct | -0.175 | 0.270 | -0.648 | 0.517 |
| Apr - Sep | -0.437 | 0.267 | -1.637 | 0.102 |
| Aug - Dec | 0.918 | 0.140 | 6.581 | <.0001 |
| Aug - Feb | 1.108 | 0.193 | 5.748 | <.0001 |
| Aug - Jan | 0.917 | 0.160 | 5.722 | <.0001 |
| Aug - Jul | -0.108 | 0.125 | -0.860 | 0.390 |
| Aug - Jun | 0.029 | 0.126 | 0.231 | 0.817 |
| Aug - Mar | 1.477 | 0.200 | 7.372 | <.0001 |
| Aug - May | 0.607 | 0.150 | 4.051 | 0.000 |
| Aug - Nov | 1.136 | 0.146 | 7.794 | <.0001 |
| Aug - Oct | 0.992 | 0.144 | 6.877 | <.0001 |
| Aug - Sep | 0.730 | 0.131 | 5.557 | <.0001 |
| Dec - Feb | 0.189 | 0.196 | 0.967 | 0.334 |
| Dec - Jan | -0.001 | 0.163 | -0.006 | 0.996 |
| Dec - Jul | -1.026 | 0.139 | -7.363 | <.0001 |
| Dec - Jun | -0.889 | 0.141 | -6.309 | <.0001 |
| Dec - Mar | 0.559 | 0.210 | 2.668 | 0.008 |
| Dec - May | -0.311 | 0.159 | -1.955 | 0.051 |
| Dec - Nov | 0.218 | 0.150 | 1.448 | 0.148 |
| Dec - Oct | 0.074 | 0.151 | 0.489 | 0.625 |
| Dec - Sep | -0.188 | 0.144 | -1.309 | 0.191 |
| Feb - Jan | -0.190 | 0.205 | -0.929 | 0.353 |
| Feb - Jul | -1.215 | 0.192 | -6.318 | <.0001 |
| Feb - Jun | -1.078 | 0.193 | -5.601 | <.0001 |
| Feb - Mar | 0.370 | 0.244 | 1.514 | 0.130 |
| Feb - May | -0.500 | 0.206 | -2.432 | 0.015 |
| Feb - Nov | 0.028 | 0.199 | 0.142 | 0.887 |
| Feb - Oct | -0.116 | 0.199 | -0.582 | 0.560 |
| Feb - Sep | -0.377 | 0.196 | -1.924 | 0.054 |
| Jan - Jul | -1.025 | 0.160 | -6.401 | <.0001 |
| Jan - Jun | -0.888 | 0.160 | -5.536 | <.0001 |
| Jan - Mar | 0.560 | 0.221 | 2.536 | 0.011 |
| Jan - May | -0.310 | 0.175 | -1.767 | 0.077 |
| Jan - Nov | 0.219 | 0.167 | 1.310 | 0.190 |
| Jan - Oct | 0.074 | 0.168 | 0.444 | 0.657 |
| Jan - Sep | -0.187 | 0.164 | -1.139 | 0.255 |
| Jul - Jun | 0.137 | 0.127 | 1.081 | 0.280 |
| Jul - Mar | 1.585 | 0.199 | 7.967 | <.0001 |
| Jul - May | 0.715 | 0.150 | 4.759 | <.0001 |
| Jul - Nov | 1.244 | 0.145 | 8.562 | <.0001 |
| Jul - Oct | 1.100 | 0.143 | 7.666 | <.0001 |
| Jul - Sep | 0.838 | 0.132 | 6.349 | <.0001 |
| Jun - Mar | 1.448 | 0.200 | 7.255 | <.0001 |
| Jun - May | 0.578 | 0.151 | 3.828 | 0.000 |
| Jun - Nov | 1.107 | 0.146 | 7.563 | <.0001 |
| Jun - Oct | 0.963 | 0.145 | 6.657 | <.0001 |
| Jun - Sep | 0.701 | 0.133 | 5.265 | <.0001 |
| Mar - May | -0.870 | 0.216 | -4.032 | 0.000 |
| Mar - Nov | -0.341 | 0.211 | -1.617 | 0.106 |
| Mar - Oct | -0.485 | 0.209 | -2.323 | 0.020 |
| Mar - Sep | -0.747 | 0.205 | -3.651 | 0.000 |
| May - Nov | 0.529 | 0.164 | 3.219 | 0.001 |
| May - Oct | 0.384 | 0.163 | 2.351 | 0.019 |
| May - Sep | 0.123 | 0.155 | 0.792 | 0.428 |
| Nov - Oct | -0.144 | 0.156 | -0.925 | 0.355 |
| Nov - Sep | -0.406 | 0.150 | -2.703 | 0.007 |
| Oct - Sep | -0.262 | 0.149 | -1.757 | 0.079 |

### Table A6. Model coefficients of Generalized Additive Mixed Model of the association between daily ranges moved by platypuses with implanted transmitters on the Mitta Mitta River, in response to month, flow, water level, rainfall and total number of detections, with an interaction term among individual platypuses and month.

| **Parametric coefficients** | **Estimate** | **S.E** | **T value** | **P** |
| --- | --- | --- | --- | --- |
| Intercept | -0.285 | 0.323 | -0.883 | 0.378 |
|  | | | | |
| **Smooth terms** | **Edf** | **Ref Df** | **F** | **P** |
| Counts | 1.944 | 1.944 | 48.919 | <0.001 |
| Month | 1.973 | 1.973 | 23.483 | <0.001 |
| Rainfall | 1.000 | 1.000 | 0.946 | 0.331 |
| Flow | 1.945 | 1.945 | 11.899 | <0.001 |
| R-sq.(adj) = 0.031 Scale est. = 0.81969 n = 928 | | | | |

### Figure A3. Generalized Additive Mixed Model plots of the average daily range movements by platypuses with implanted transmitters on the Mitta Mitta River.

*
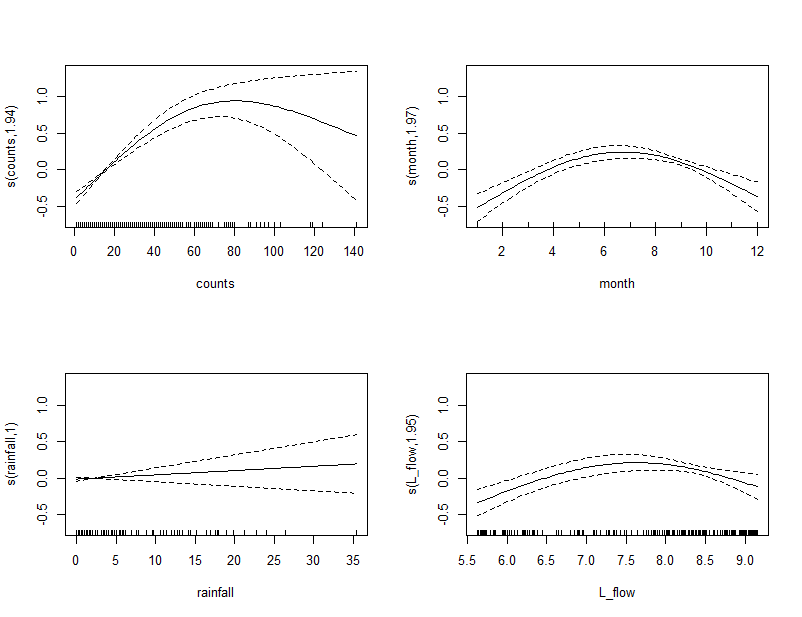
*

## Appendix 3. Models for cumulative movements

### Table A7. Model coefficients of the Generalized Mixed Effect Model of daily cumulative movements by platypuses with implanted transmitters on the Snowy River across months with an interaction between month and sex (Mar-Jul 2017).

| **Fixed effects** | **Estimate** | **Std. Error** | **t value** | **P** |
| --- | --- | --- | --- | --- |
| (Intercept) | -0.227 | 0.298 | -0.762 | 0.446 |
| July | -1.194 | 0.164 | -7.294 | <0.001 |
| June | -1.412 | 0.162 | -8.722 | <0.001 |
| March | -0.357 | 0.149 | -2.395 | 0.017 |
| May | -0.866 | 0.156 | -5.550 | <0.001 |
| Male | 0.246 | 0.422 | 0.582 | 0.560 |
| July:Male | 1.675 | 0.248 | 6.747 | <0.001 |
| June:Male | 1.518 | 0.249 | 6.097 | <0.001 |
| March:Male | 0.513 | 0.236 | 2.176 | 0.030 |
| May:Male | 0.973 | 0.226 | 4.302 | <0.001 |
| AIC BIC logLik deviance df.resid  916.3 971.8 -446.2 892.3 741 | | | | |

### Table A8. Post-hoc tests based on estimated marginal means of a Generalized Mixed Effect Model of cumulative distances moved by platypuses with implanted transmitters on the Snowy River.

| **Contrast** | **Estimate** | **SE** | **z.ratio** | **p.value** |
| --- | --- | --- | --- | --- |
| Apr Female - Jul Female | 1.190 | 0.164 | 7.294 | <.0001 |
| Apr Female - Jun Female | 1.410 | 0.162 | 8.722 | <.0001 |
| Apr Female - Mar Female | 0.357 | 0.149 | 2.395 | 0.017 |
| Apr Female - May Female | 0.866 | 0.156 | 5.55 | <.0001 |
| Apr Female - Apr Male | -0.246 | 0.422 | -0.582 | 0.560 |
| Apr Female - Jul Male | -0.728 | 0.432 | -1.684 | 0.092 |
| Apr Female - Jun Male | -0.352 | 0.434 | -0.811 | 0.418 |
| Apr Female - Mar Male | -0.402 | 0.431 | -0.932 | 0.351 |
| Apr Female - May Male | -0.352 | 0.424 | -0.831 | 0.406 |
| Jul Female - Jun Female | 0.218 | 0.156 | 1.4 | 0.162 |
| Jul Female - Mar Female | -0.836 | 0.163 | -5.139 | <.0001 |
| Jul Female - May Female | -0.327 | 0.154 | -2.128 | 0.033 |
| Jul Female - Apr Male | -1.440 | 0.423 | -3.401 | 0.001 |
| Jul Female - Jul Male | -1.920 | 0.433 | -4.439 | <.0001 |
| Jul Female - Jun Male | -1.550 | 0.435 | -3.552 | 0.000 |
| Jul Female - Mar Male | -1.600 | 0.431 | -3.697 | 0.000 |
| Jul Female - May Male | -1.550 | 0.425 | -3.638 | 0.000 |
| Jun Female - Mar Female | -1.050 | 0.161 | -6.548 | <.0001 |
| Jun Female - May Female | -0.545 | 0.153 | -3.574 | 0.000 |
| Jun Female - Apr Male | -1.660 | 0.423 | -3.92 | 0.000 |
| Jun Female - Jul Male | -2.140 | 0.432 | -4.947 | <.0001 |
| Jun Female - Jun Male | -1.760 | 0.435 | -4.057 | <.0001 |
| Jun Female - Mar Male | -1.810 | 0.431 | -4.207 | <.0001 |
| Jun Female - May Male | -1.760 | 0.424 | -4.156 | <.0001 |
| Mar Female - May Female | 0.509 | 0.155 | 3.277 | 0.001 |
| Mar Female - Apr Male | -0.603 | 0.422 | -1.429 | 0.153 |
| Mar Female - Jul Male | -1.080 | 0.432 | -2.512 | 0.012 |
| Mar Female - Jun Male | -0.709 | 0.434 | -1.633 | 0.102 |
| Mar Female - Mar Male | -0.759 | 0.43 | -1.762 | 0.078 |
| Mar Female - May Male | -0.709 | 0.424 | -1.674 | 0.094 |
| May Female - Apr Male | -1.110 | 0.421 | -2.641 | 0.008 |
| May Female - Jul Male | -1.590 | 0.431 | -3.7 | 0.000 |
| May Female - Jun Male | -1.220 | 0.433 | -2.812 | 0.005 |
| May Female - Mar Male | -1.270 | 0.429 | -2.952 | 0.003 |
| May Female - May Male | -1.220 | 0.423 | -2.882 | 0.004 |
| Apr Male - Jul Male | -0.482 | 0.187 | -2.575 | 0.010 |
| Apr Male - Jun Male | -0.106 | 0.19 | -0.56 | 0.575 |
| Apr Male - Mar Male | -0.156 | 0.182 | -0.853 | 0.394 |
| Apr Male - May Male | -0.106 | 0.163 | -0.65 | 0.516 |
| Jul Male - Jun Male | 0.376 | 0.2 | 1.879 | 0.060 |
| Jul Male - Mar Male | 0.326 | 0.189 | 1.722 | 0.085 |
| Jul Male - May Male | 0.376 | 0.196 | 1.916 | 0.055 |
| Jun Male - Mar Male | -0.049 | 0.197 | -0.251 | 0.802 |
| Jun Male - May Male | 0.000 | 0.197 | 0 | 1.000 |
| Mar Male - May Male | 0.049 | 0.191 | 0.259 | 0.796 |

### Table A9. Model coefficients of Generalized Additive Mixed Model of the association between daily cumulative movements by platypuses with implanted transmitters on the Snowy River in response to month, flow and total number of detections, with an interaction term among individual platypuses and month.

| **Parametric coefficients** | **Estimate** | **S.E** | **T value** | **P** |
| --- | --- | --- | --- | --- |
| (Intercept) | -0.543 | 0.236 | -2.296 | 0.022 |
|  | | | | |
| **Smooth terms** | **Edf** | **Ref Df** | **F** | **P** |
| Counts | 1.952 | 1.952 | 21.398 | <0.001 |
| Month:Female | 1.000 | 1.000 | 21.542 | <0.001 |
| Month:Male | 1.741 | 1.741 | 24.247 | <0.001 |
| Rainfall | 1.000 | 1.000 | 0.052 | 0.820 |
| Flow | 1.109 | 1.109 | 4.254 | 0.029 |
| R-sq.(adj) = 0.0795 Scale est. = 1.2096 n = 875 | | | | |

### Figure A4. Generalized Additive Mixed Model plots of the daily cumulative movements by platypuses with implanted transmitters on the Snowy River.

*
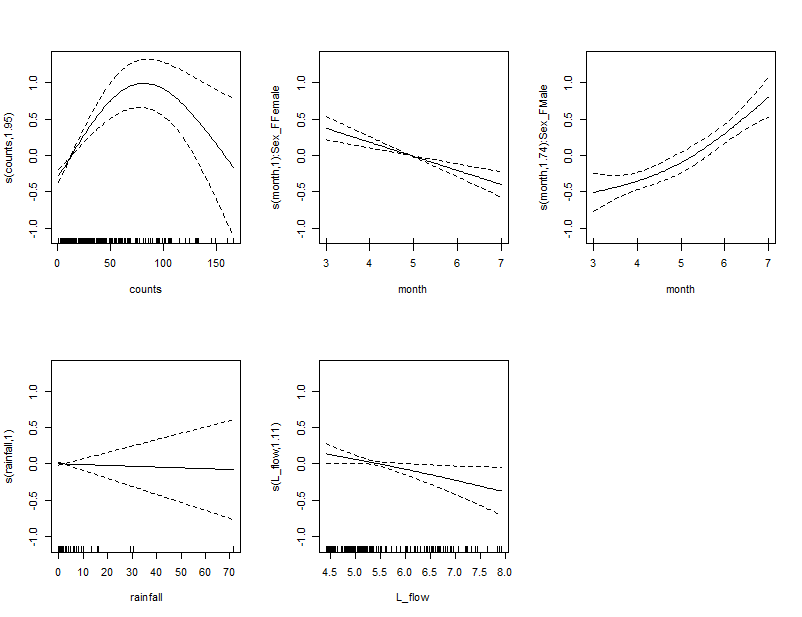
*

### Table A10. Model coefficients of the Generalized Mixed Effect Model of daily cumulative movements by platypuses with on the Mitta Mitta River across months (May 2018-Apri 2019).

| **Fixed effects** | **Estimate** | **Std. Error** | **t value** | **P** |
| --- | --- | --- | --- | --- |
| (Intercept) | 0.151 | 0.383 | 0.394 | 0.694 |
| August | 1.161 | 0.241 | 4.826 | <0.001 |
| December | -0.065 | 0.249 | -0.262 | 0.794 |
| February | -0.201 | 0.275 | -0.733 | 0.464 |
| January | 0.105 | 0.258 | 0.405 | 0.685 |
| July | 1.062 | 0.240 | 4.420 | <0.001 |
| June | 0.865 | 0.240 | 3.596 | <0.001 |
| March | -0.202 | 0.274 | -0.739 | 0.460 |
| May | 0.296 | 0.252 | 1.173 | 0.241 |
| November | -0.080 | 0.251 | -0.318 | 0.750 |
| October | 0.125 | 0.248 | 0.506 | 0.613 |
| September | 0.404 | 0.244 | 1.657 | 0.097 |
| AIC BIC logLik deviance df.resid  2869.7 2937.1 -1420.9 2841.7 89 | | | | |

### Table A11. Post-hoc tests based on estimated marginal means of a Generalized Mixed Effect Model of cumulative distances moved by platypuses with implanted transmitters on the Mitta Mitta River.

| **Contrast** | **Estimate** | **SE** | **z.ratio** | **p.value** |
| --- | --- | --- | --- | --- |
| Apr - Aug | -1.161 | 0.241 | -4.826 | <.0001 |
| Apr - Dec | 0.065 | 0.249 | 0.262 | 0.794 |
| Apr - Feb | 0.201 | 0.275 | 0.733 | 0.464 |
| Apr - Jan | -0.105 | 0.258 | -0.405 | 0.685 |
| Apr - Jul | -1.062 | 0.240 | -4.420 | <.0001 |
| Apr - Jun | -0.865 | 0.240 | -3.596 | 0.000 |
| Apr - Mar | 0.202 | 0.274 | 0.739 | 0.460 |
| Apr - May | -0.296 | 0.252 | -1.173 | 0.241 |
| Apr - Nov | 0.080 | 0.251 | 0.318 | 0.750 |
| Apr - Oct | -0.125 | 0.248 | -0.506 | 0.613 |
| Apr - Sep | -0.404 | 0.244 | -1.657 | 0.097 |
| Aug - Dec | 1.226 | 0.130 | 9.453 | <.0001 |
| Aug - Feb | 1.362 | 0.177 | 7.678 | <.0001 |
| Aug - Jan | 1.056 | 0.149 | 7.106 | <.0001 |
| Aug - Jul | 0.099 | 0.115 | 0.865 | 0.387 |
| Aug - Jun | 0.296 | 0.116 | 2.559 | 0.011 |
| Aug - Mar | 1.363 | 0.183 | 7.466 | <.0001 |
| Aug - May | 0.865 | 0.138 | 6.272 | <.0001 |
| Aug - Nov | 1.241 | 0.135 | 9.207 | <.0001 |
| Aug - Oct | 1.036 | 0.133 | 7.810 | <.0001 |
| Aug - Sep | 0.757 | 0.121 | 6.255 | <.0001 |
| Dec - Feb | 0.136 | 0.179 | 0.759 | 0.448 |
| Dec - Jan | -0.170 | 0.149 | -1.140 | 0.254 |
| Dec - Jul | -1.127 | 0.128 | -8.775 | <.0001 |
| Dec - Jun | -0.930 | 0.130 | -7.182 | <.0001 |
| Dec - Mar | 0.137 | 0.191 | 0.717 | 0.474 |
| Dec - May | -0.361 | 0.146 | -2.480 | 0.013 |
| Dec - Nov | 0.015 | 0.137 | 0.106 | 0.916 |
| Dec - Oct | -0.191 | 0.138 | -1.380 | 0.168 |
| Dec - Sep | -0.470 | 0.133 | -3.544 | 0.000 |
| Feb - Jan | -0.306 | 0.188 | -1.631 | 0.103 |
| Feb - Jul | -1.263 | 0.177 | -7.136 | <.0001 |
| Feb - Jun | -1.066 | 0.177 | -6.024 | <.0001 |
| Feb - Mar | 0.001 | 0.223 | 0.004 | 0.997 |
| Feb - May | -0.497 | 0.189 | -2.636 | 0.008 |
| Feb - Nov | -0.122 | 0.183 | -0.666 | 0.505 |
| Feb - Oct | -0.327 | 0.183 | -1.789 | 0.074 |
| Feb - Sep | -0.606 | 0.179 | -3.375 | 0.001 |
| Jan - Jul | -0.957 | 0.148 | -6.464 | <.0001 |
| Jan - Jun | -0.760 | 0.148 | -5.133 | <.0001 |
| Jan - Mar | 0.307 | 0.201 | 1.526 | 0.127 |
| Jan - May | -0.191 | 0.161 | -1.186 | 0.236 |
| Jan - Nov | 0.184 | 0.153 | 1.202 | 0.229 |
| Jan - Oct | -0.021 | 0.154 | -0.134 | 0.894 |
| Jan - Sep | -0.300 | 0.151 | -1.989 | 0.047 |
| Jul - Jun | 0.197 | 0.116 | 1.697 | 0.090 |
| Jul - Mar | 1.264 | 0.182 | 6.941 | <.0001 |
| Jul - May | 0.766 | 0.138 | 5.561 | <.0001 |
| Jul - Nov | 1.141 | 0.134 | 8.538 | <.0001 |
| Jul - Oct | 0.936 | 0.132 | 7.117 | <.0001 |
| Jul - Sep | 0.657 | 0.121 | 5.424 | <.0001 |
| Jun - Mar | 1.067 | 0.182 | 5.852 | <.0001 |
| Jun - May | 0.569 | 0.138 | 4.112 | <.0001 |
| Jun - Nov | 0.945 | 0.134 | 7.024 | <.0001 |
| Jun - Oct | 0.740 | 0.132 | 5.588 | <.0001 |
| Jun - Sep | 0.460 | 0.122 | 3.773 | 0.000 |
| Mar - May | -0.498 | 0.196 | -2.537 | 0.011 |
| Mar - Nov | -0.122 | 0.193 | -0.634 | 0.526 |
| Mar - Oct | -0.327 | 0.191 | -1.719 | 0.086 |
| Mar - Sep | -0.607 | 0.186 | -3.257 | 0.001 |
| May - Nov | 0.376 | 0.150 | 2.499 | 0.013 |
| May - Oct | 0.171 | 0.149 | 1.142 | 0.254 |
| May - Sep | -0.109 | 0.142 | -0.763 | 0.445 |
| Nov - Oct | -0.205 | 0.143 | -1.434 | 0.151 |
| Nov - Sep | -0.484 | 0.138 | -3.515 | 0.000 |
| Oct - Sep | -0.279 | 0.136 | -2.048 | 0.041 |

### Table A12. Model coefficients of Generalized Additive Mixed Model of the association between daily cumulative movements by platypuses on the Mitta Mitta River, in response to month, flow and total number of detections, with an interaction term among individual platypuses and month.

| **Parametric coefficients** | **Estimate** | **S.E** | **T value** | **P** |
| --- | --- | --- | --- | --- |
| (Intercept) | 0.613 | 0.318 | 1.872 | 0.062 |
|  | | | | |
| **Smooth terms** | **Edf** | **Ref Df** | **F** | **P** |
| Counts | 1.964 | 1.964 | 61.704 | <0.001 |
| Month | 1.988 | 1.988 | 47.042 | <0.001 |
| Rainfall | 1.000 | 1.000 | 5.235 | 0.022 |
| Flow | 1.917 | 1.917 | 24.124 | <0.001 |
| R-sq.(adj) = 0.12 Scale est. = 0.59959 n = 910 | | | | |

### Figure A5. Generalized Additive Mixed Model plots of the daily cumulative movements by platypuses with implanted transmitters on the Mitta Mitta River.


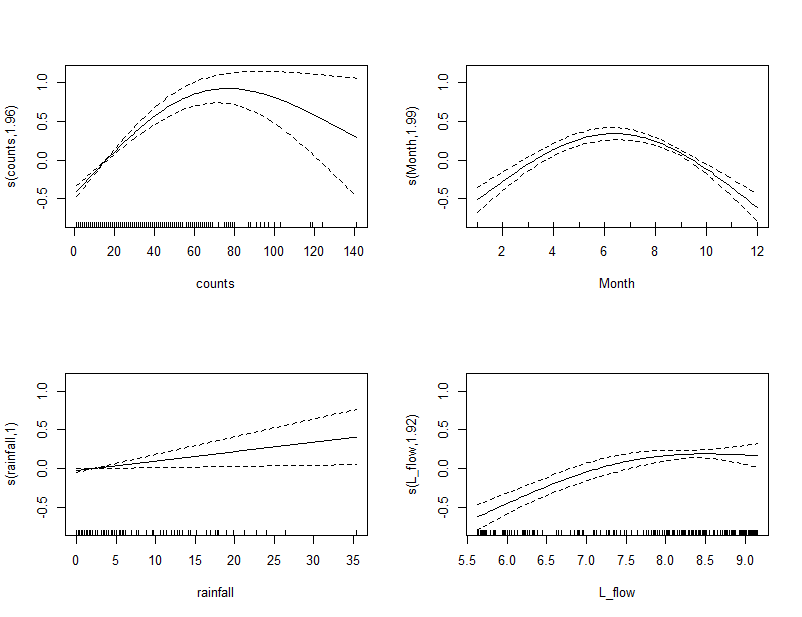

Supplement: Supplementary file 1 — Supplementary Information. [file 41598_2021_81142_MOESM1_ESM.docx]
